# Supplementary figures and images for: Bacteria use structural imperfect mimicry to hijack the host interactome
Source: PLoS Comput Biol. 2020 Dec 4;16(12):e1008395. doi: 10.1371/journal.pcbi.1008395 (PMC7744059; doi:10.1371/journal.pcbi.1008395)

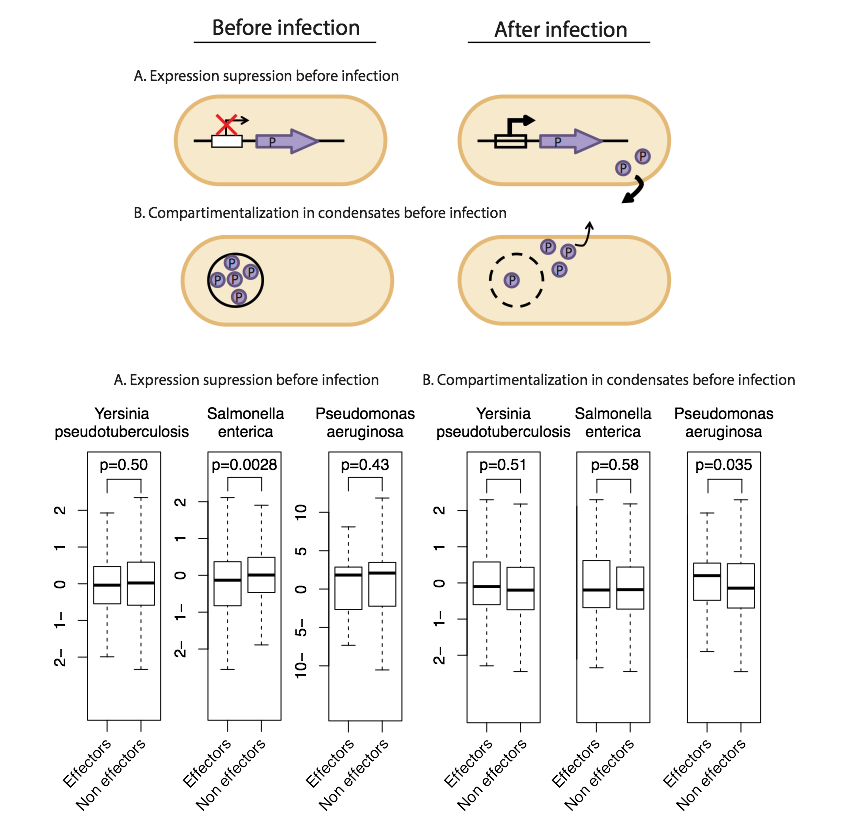

Supplement: S1 Fig — Pathogens could (A) regulate the expression of protein effectors or (B) compartmentalize the interactions in protein condensates for timely delivery upon infection. As observed in the boxplots in the lower panel, no clear significant differences were observed in all three organisms studied. P-values were calculated using the Mann-Whitney U test for comparing pairs of independent samples. (TIFF) [file pcbi.1008395.s001.tiff]

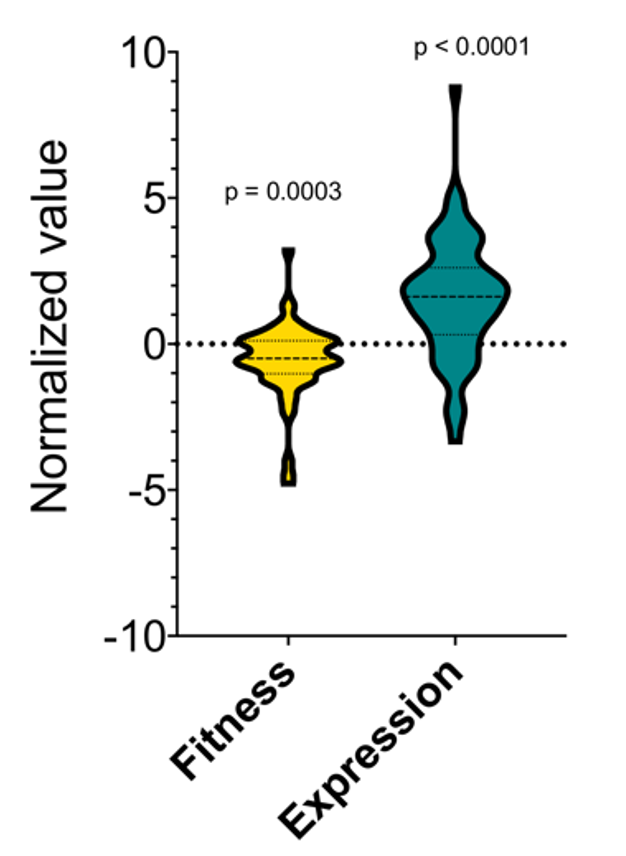

Supplement: S2 Fig — To validate the role of bacterial proteins in the BE dataset during infection, we used blastp against the BacFitBase database (http://www.tartaglialab.com/bacfitbase/) to predict the fitness score associated with each protein. The fitness defect associated to these proteins is larger than a null effect (p = 0.0005, negative values indicate a reduction on pathogen fitness), suggesting that the BE dataset is associated with pathogenesis. Also, we analyzed the mRNA expression data using the DualSeqDB database (http://www.tartaglialab.com/dualseq/). Proteins belonging to the BE dataset are significantly upregulated in infection (p<0.0001) suggesting its implication in bacterial pathogenesis. (TIFF) [file pcbi.1008395.s002.tiff]

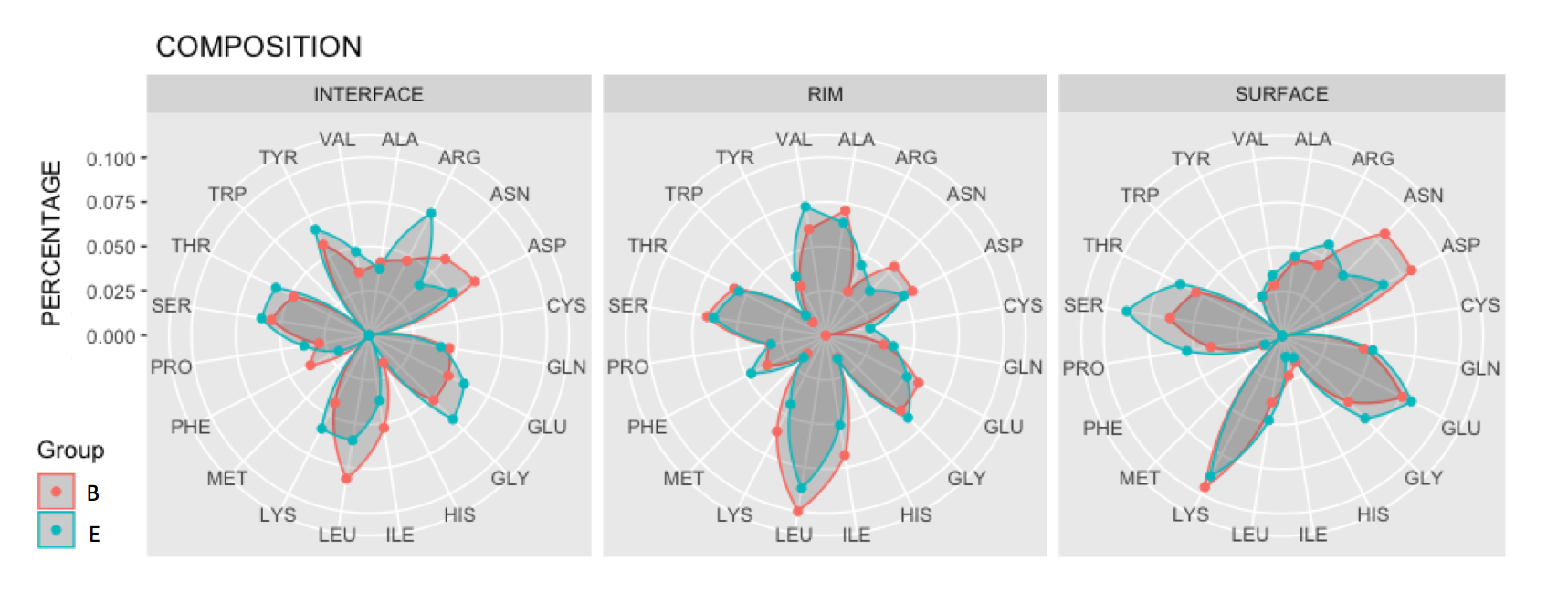

Supplement: S3 Fig — Percentage of each amino acid in the interface, rim and surface regions of bacteria-eukaryote complexes. Each region is subdivided in bacteria (B, red) and eukaryote counterparts (E, blue). (TIFF) [file pcbi.1008395.s003.tiff]

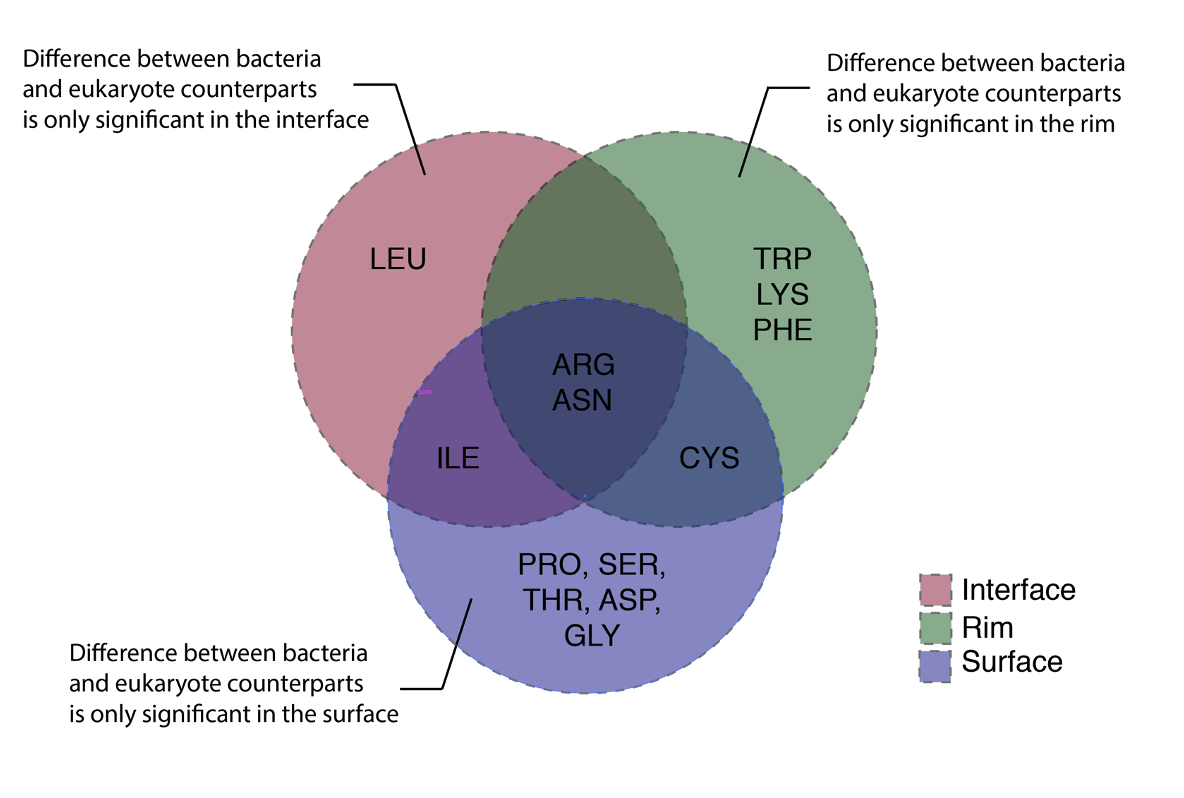

Supplement: S4 Fig — Venn diagram displaying the differential amino acid composition of interface, rim and surface areas in bacteria-eukaryote complexes. Significant amino acid composition differences between bacteria and eukaryote counterparts in bacteria-eukaryote complexes were calculated using a Mann-Whitney U test and considered significant when p < 0.05. (TIFF) [file pcbi.1008395.s004.tiff]

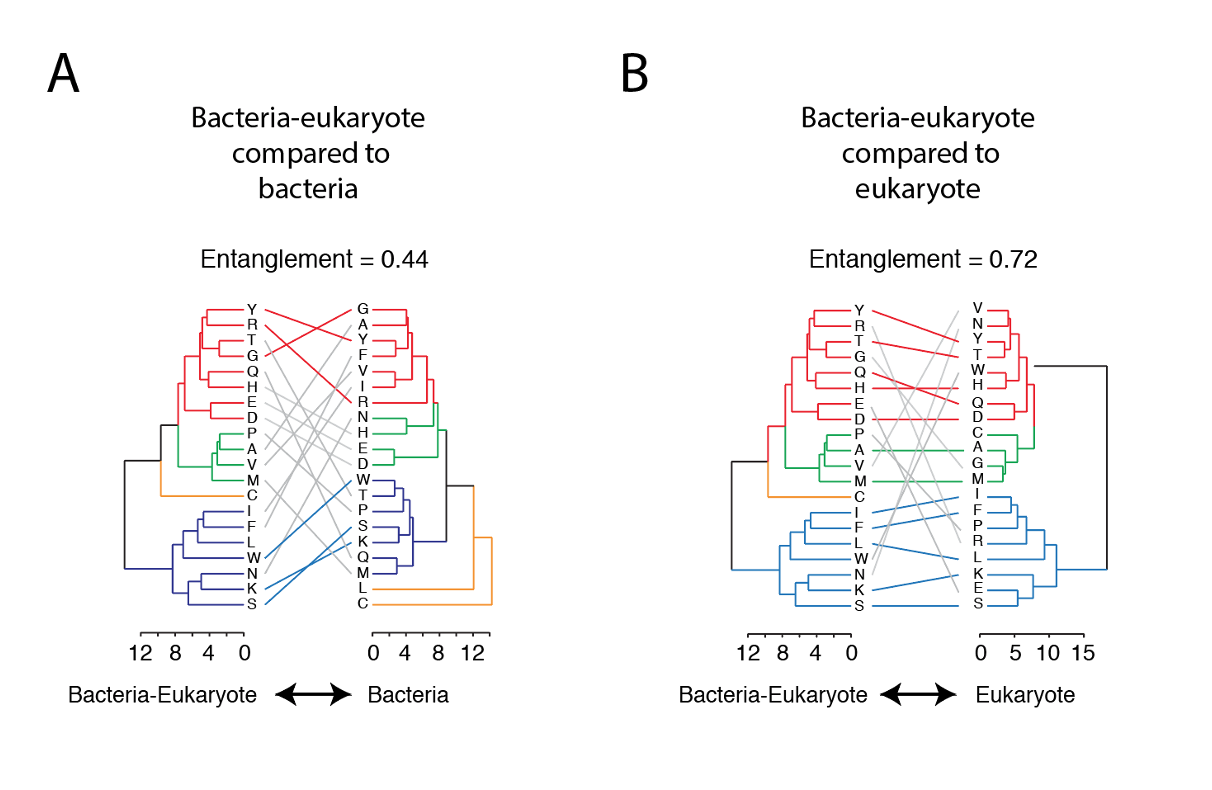

Supplement: S5 Fig — Dendrograms for each complex type (bacteria, eukaryote and bacteria-eukaryote) were built according to the interaction pattern of each amino acid using the Ward’s minimum variance method. For each dendrogram, four groups (red, green, orange and blue) were defined using k-means clustering. Then, tanglegrams were built to compare the congruence between dendrograms (bacteria-eukaryote compared to bacteria and eukaryote). In the figure, congruence is depicted by the number of colored lines mapping common elements between same groups in different dendrograms. The quality of the alignment of the two dendrograms (entanglement) is also reported as a quantitative measure of congruence. Entanglement is measured between 1 (full entanglement, high congruence) to 0 (no entanglement, null congruence). Tanglegrams were built using the dendextend package in R. (TIFF) [file pcbi.1008395.s005.tiff]

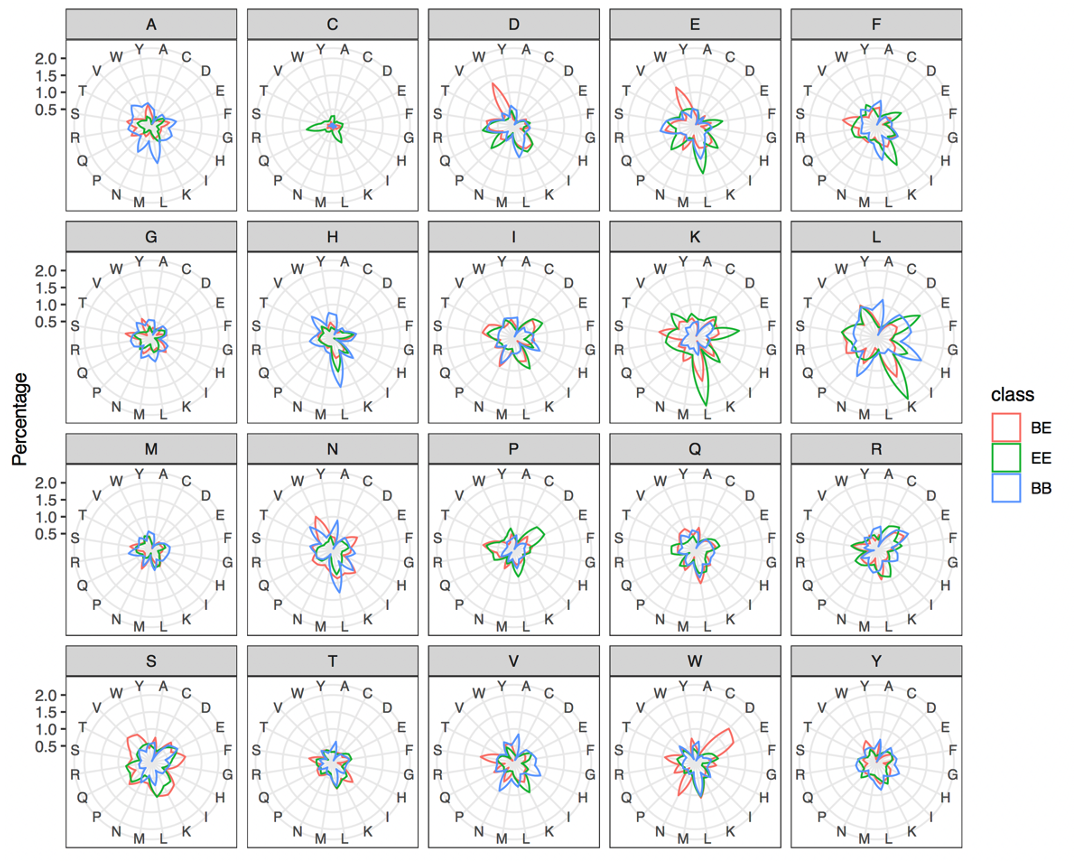

Supplement: S6 Fig — Number of interactions for each amino acid (measured as the percentage relative to the total number of interactions) in bacteria-eukaryote (BE, red), bacteria (BB, blue) and eukaryote (EE, green) complexes. Each pair of letters correspond to an amino acid in the interaction pair. The scale measures the percentage of a given interaction over all detected interactions. (TIFF) [file pcbi.1008395.s006.tiff]

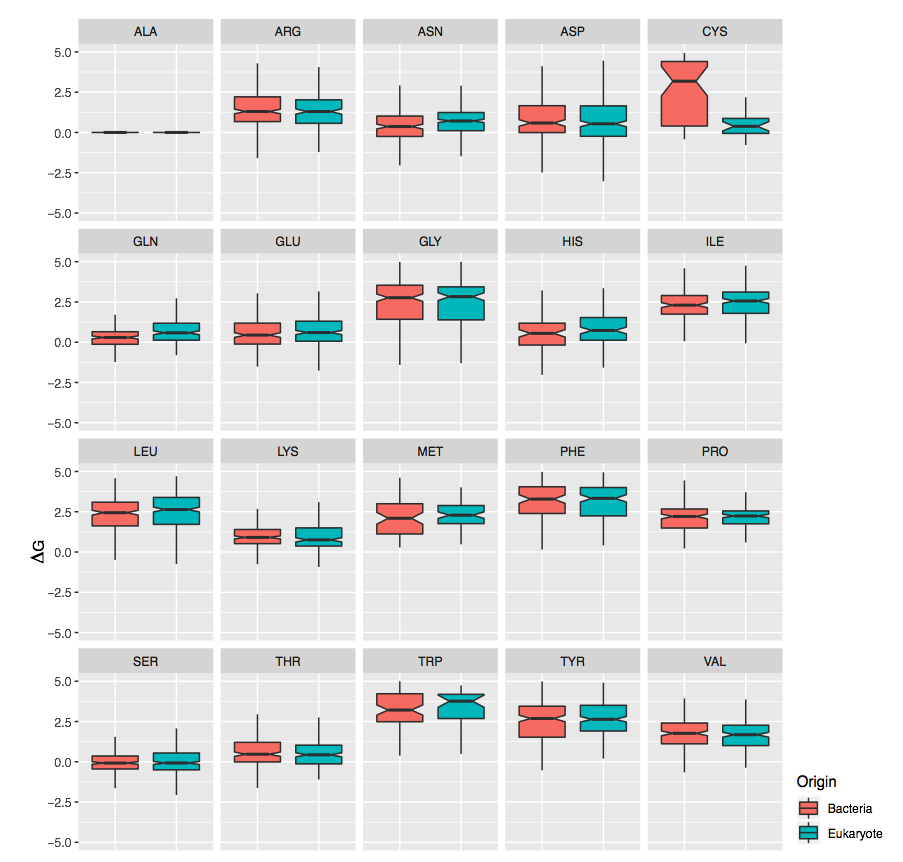

Supplement: S7 Fig — Each residue in the interface of bacteria-eukaryote complexes was mutated to alanine and the change in complex stability was calculated using FoldX. The impact in complex stability for mutations in bacteria (red) and eukaryote (blue) interaction counterparts is compared. (TIFF) [file pcbi.1008395.s007.tiff]
