# Supplementary material for: Bacteria use structural imperfect mimicry to hijack the host interactome
Source: PLoS Comput Biol. 2020 Dec 4;16(12):e1008395. doi: 10.1371/journal.pcbi.1008395 (PMC7744059; doi:10.1371/journal.pcbi.1008395)
Supplement: Attachment — Submitted filename: Point_by_point_response.pdf [file pcbi.1008395.s011.pdf]

Point-by-point response

Reviewer #1

The manuscript "Bacteria use Structural imperfect mimicry to hijack the host interactome" examines host-pathogen interactome networks and pathogen interactome networks, and finds central hub proteins in host-pathogen interactomes are less connected in the pathogen interactomes. The paper then separates interaction sites into core and rim interaction regions (solvent-excluding central areas of interaction and surrounding regions, respectively) and examines amino acid biases in each region across interaction types (within pathogen, within host and between host and pathogen) in an attempt to identify protein structural or compositional trends that may explain binding, and that in terms of amino acid residue interactions, bacteria-eukaryote complexes had more in common with eukaryotic complexes than bacterial complexes. Lastly, the paper postulates an evolutionary balance of pressures between increased binding propensity to host proteins by mimicking eukaryotic 'stickier' binding sites, and limiting potentially fitness-disruptive self-binding interactions, resulting in imperfect mimicry.

The study is interesting, and an understanding of broad structural or evolutionary trends in host-pathogen interactions is timely. The difference in network hub properties and the suggestion that bacteria-eukaryotic complexes more closely mimic eukaryotic complexes is intriguing.

We thank the reviewer for considering our paper timely and of broad interest to the scientific community. The comments were very constructive and helped us to improve the manuscript. We hope that the revised version will meet the expectation of this reviewer for publication.

1. As data is mined from the PDB and Uniprot, I would like to see some discussion of the limitations and potential biases of this dataset, especially as eukaryote-bacterial interactions are conceptually an enormous potential data set and also relatively new and limited compared to eukaryotic or even bacterial protein interaction networks. This limitation is reflected in the data used, which examines almost 700 eukaryotic complexes but only 90 bacteria-eukaryote complexes.

We acknowledge that bacteria-eukaryote (EB) and bacteria-bacteria (BB) datasets are smaller compared to the eukaryote (EE) set. Of course, this reflects a higher research intensity on characterizing EE complexes. To verify that the similarity between BE and EE complexes depends neither on the nature of proteins included nor in its sample size, we conducted two control calculations:

i. We compared the interface size in all sets, measured as the number of interactions per molecule. As shown in Figure R1A, all complexes have a similar number of interactions per molecule and the differences observed between sets are not significant ($p > 0.05$).

ii. We used resampling to create balanced datasets and determine the effect of sample size. To generate balanced datasets, we randomly undersampled the majority class (EE complexes) and oversampled the minority class (EB complexes). Using this strategy, we simulated 500 balanced datasets and repeated the calculations to determine whether there is a significant difference in amino acid connectivity, as shown in Figure 2F. We found that differences in connectivity are still significant after resampling, with an average p-value of 0.023 (Figure R1B). This result is consistent with the value reported previously. Hence, the results are robust under resampling, reinforcing the conclusions presented in the manuscript.

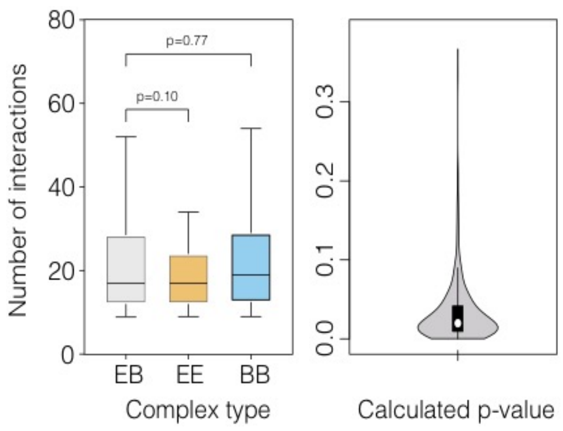

Figure R1. ***Control calculations in the PDB complex sets.*** (A) Number of interactions per molecule in EB, EE and BB complexes. (B) Sample size effect measured as the p-value distribution in balanced datasets.

We have also added a detailed description of the limitations of our study. Specifically, we discuss how we controlled the potential biases (Pages 8-9) and how the robustness analyses suggest that our conclusions are sound (updated Figure 2 panels B and F and legend).

If I understand correctly, molecular mimicry suggests similarity in protein structure architecture or shape between a pathogenic protein and a host protein it 'mimics' results in pathogenic binding. However, binding can be due to a small number of amino acids. Mere amino acid composition at the level of a negatively-charged amino acid will interact with positively charged one does not seem sufficient to argue for structural mimicry beyond the most basic of biochemical properties. An examination of short sequences and their homologies would be more convincing.

The reviewer is right pointing out that amino acid composition is not enough to characterize binding. In fact, we show in Figure 2B and 2C that composition itself is very similar between BE, EE and BB complexes. Interfaces span over a large area (usually >2000 Å; Jones S. and Thornton J.M., 1996, *PNAS* 93:13–20) and are often discontinuous (Tuncbag N. et al., 2008, *J. Mol. Biol.* 381:785–802). Also, interfaces are not constrained to single sequences, but short stretches of amino acids that may be far apart in the structure. Hence, the complexity of these interactions is difficult to assess using sequence homology.
We use the term structural mimicry to refer not only to the composition of the interface but to its geometry and interactions. Hence, instead of composition, we use connectivity to compare complexes. We think of connectivity profiles as interaction fingerprints shaped by the evolutionary history of the host and pathogen. Our results (Figure 1, panels D-F) support these conclusions. Also, in our *Y. pestis* model, BE domain-domain interactions are more similar to EE complexes than BB complexes, according to Figure 1D.

To illustrate the above reasoning with an example, we selected the well-studied complex between the human T cell receptor beta chain and exotoxin A (SpeA) from *Streptococcus pyogenes* (complex 1L0Y, included in our dataset). The exotoxin contains a small segment NLQN, located in a short alpha-helix, that interacts with a long helix of the T cell receptor. This small helix strongly contributes to binding, together with other regions in the toxin. An overall sequence similarity search can only find exotoxins from other species (>70% similarity). A restricted search to the helical fragment containing the NLQL sequence, only finds exotoxins from other Streptococcus species (E-value threshold 0.01). However, by using structural similarity (Siena server: https://proteinsplus.zbh.uni-hamburg.de) we could find other effectors including SidK from Legionella pneumophila. By restricting the search to the NLQL region, we also found a similar structure in the Apical membrane antigen 1 (AMA1) from *Plasmodium falciparum*, involved in the invasion of erythrocytes. This example shows that sequence similarity is not able to recapitulate the complexity of protein interfaces and only structural-based comparisons are reliable.

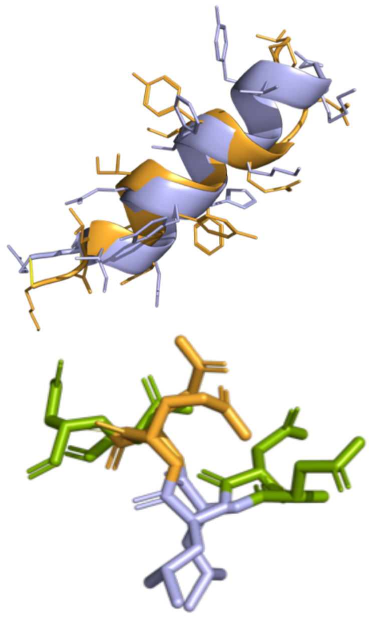

Figure R2. **Structural comparison of exotoxin A.** Structural similarity in the interaction helix (top) and NLQL segment (bottom) with protein SidK from *Legionella pneumophila.*

2 . In order to validate the similarity of BE (pathogen and host interactome) to EE, the significance of correlation between the network of interactions for each amino acid of the non-pathogenic bacteria and eukaryotic interactome should be compared. As those interaction might not be specific with respect to host pathology.

We thank the reviewer for this interesting suggestion. Indeed, comparing the network of interactions between pathogenic and non-pathogenic bacteria would be extremely interesting. However, as noted before by the reviewer, the number of BE structures in the PDB database is limited. Even if we found a handful of structures, the results obtained would not have statistical meaning. Besides, the number of validated interactions for non-pathogenic microorganisms is scarce, hindering any attempt of computational modelling. Due to these limitations, we conducted an experiment in *Y. pestis* (Figure 3), to assess how our observations would scale with an orthogonal dataset of a pathogenic microorganism. Though modelling needs to be taken with caution, our results suggest that our conclusions are sound.

3 . It would be helpful for the readers to understand the limitations involved in their data analysis pipeline.

We have extended the *Databases* section in Materials and Methods (Pages 8-9) to summarize the limitations of our study and how we controlled for potential biases in the datasets. We have also integrated Figures R1B and R3 in the manuscript (Figure 2F and 1A, respectively) and added a list of all proteins included in the cluster analysis (Supplementary Table S3).

Minor concerns:
1. The first part of the results section properly belongs in the introduction, as it provides background information.
We have moved the first paragraph of the results to the introduction section.

2. The manuscript could use a minor grammatical pass.
We have edited and corrected minor grammatical issues.

3. Numbers of complexes do not match between figure 2 and figure 2 caption.
We apologize for the mistake; we have corrected the figure legend accordingly.

4. In Figure S5, what are the alphabet in each of the panel represent as there is no description provided in the figure legend.
We thank the reviewer for this comment. We have extended the figure legend to include this point.

5. Figure 3D is hard to understand.
Figure 3D is equivalent to 1F, as calculated for *Y. pestis-H. sapiens* interactome. We acknowledge that the figure legend was too short. We have extended the text to improve clarity.

Reviewer #2

In the article titled "Bacteria use structural imperfect mimicry to hijack the host interactome" the authors explore the interactions between bacterial proteins and the host proteome. The article is generally well written and raising some interesting points however I have some concerns regarding the underly hypothesis and conclusions.

We thank the reviewer for considering that our work is of interest in the field of bacterial infection. As suggested by the reviewer, we validated our conclusions with additional datasets, when possible, and added a discussion about the limitations and potential biases of our study. Also, we performed additional tests to assess the robustness of our results. We hope that, with all these changes, the reviewer will consider that our manuscript is suitable for publication.

The central concept of the manuscript is that pathogens need to "minimize toxicity" by limiting interactions of effector proteins within the pathogen. While I agree that the bacteria likely wants to limit interactions between bacterial effector proteins and the rest of the bacterial proteome, and that bacterial lack some of the tricks (such as temporal expression or compartmentalization) used by the host cell, the evidence presented by the authors didn't convince me that this should be a difficult challenge. The authors state that "the number of protein folds and interface geometries is limited and these structures are frequently reused", however it is hard to imagine that this is truly limiting for the bacteria. For example, iPfam (version 1.0) contains >9500 domain-domain (Finn et al., 2014 Nucleic Acids Research Vol42 pD364), and 3did (ver. 2019_01) (https://3did.irbbarcelona.org/statistics.php) contains over 13,000 domain-domain interactions. With this wealth of domain-domain interactions it is hard to agree that the bacterial would be unable to find unique interactions to use for effector protein-host interactions, which would not be required for other interactions of the bacterial proteome. In summary, while I agree that it is likely effectors proteins have limited interactions with the rest of the bacterial proteome, I think the reasoning put forward by the authors is not convincing.

We thank the reviewer for the thorough discussion of this point. We agree that many domain-domain interactions are available in nature and bacteria have a wealthy subset of interactions to test. With that sentence, we did not mean that bacteria are unable to find unique interactions. Instead, we argue that bacteria have several restrictions in the repertoire:

1. The protein interaction space is limited (Aloy P. and Russell R.B., 2004, *Nat. Biotechnol*. 22:317-21), meaning that only certain domain-domain pairs are allowed. This sets an upper limit of diversity. For example, the 3did database reports 8.328 unique domain-domain interactions, while the number of pfam domains is 14.831 (as described in Mosca R. et al., 2014, *Nucleic Acids Res*. 42:D1, D374-9).
2. The distribution of folds and domains in bacteria and eukaryotes is different. The most common folds in prokaryotes are those involved in housekeeping functions, such as P-loop-containing NTPases and TIM barrels, whereas the eukaryotic distribution is dominated by domains with regulatory functions, such as protein kinases and β-propellers (See, for example, Wolf Y.I. et al., 1999, *Genome Res* 9:17-26). This also imposes restrictions in the interaction space available.

Even in the case where a domain pair is available, the bacteria interface should adapt to properly bind the human target. We argue that, in this process, bacteria must find a balance between the least toxicity and the largest binding. Mutations at PPI interfaces, particularly at the core region, may cause large geometrical and conformational changes at the interaction sites. These events can modify the interface stability, affecting the complex assembly (Jubb H.C. et al., 2017, *Biophys. Mol. Biol.* 128:3-13). This is a general trait observed from virus (Rincon V. et al., 2015, *J. Gen. Virol*. 96:2595-606; Pappalardo M. et al., 2016, *Sci. Rep* 6:23743), to humans (Yates C.M. et al., 2013, *J. Mol. Biol*. 425:3949-63; David A. et al., 2012, *Hum. Mutat*. 33:359–363). Such changes will likely promote unspecific binding or even protein aggregation, where other proteins can be sequestered during the formation of inclusion bodies.

We acknowledge that we may have not discussed this topic with enough detail in the manuscript and the sentence mentioned by the reviewer could be expanded for clarity. We have summarized the points described above in the manuscript (Page 2). Please note that, as requested by Reviewer 1, we have moved this section to the introduction.

To explore their hypothesis the authors first looked at whether effectors were less likely to be hubs in the pathogen network. It is unclear what the source data for this analysis was, and how it may have been impacted by study bias.

We used the IntAct database to retrieve the list of host-pathogen interactions. In order to get a homogeneous set of data, we used only the interactions published in a single study (Dyer et al., 2010, *PLoS One* 5:e12089). Hence, the data is as homogeneous as possible. Then, we used the EffectiveDB database (http://effectors.org/) to classify the entries as effectors or non-effectors. The stringency criterion to discriminate between these two classes was set to 0.95. Using a different stringency threshold (0.90 to 0.99) did not change our results and all comparisons remain statistically significant at $p<0.05$. This suggests that our observations are robust and are not affected by classification stringency. We expanded the Materials and Methods section to better describe the criteria and added a sentence in Figure 1A legend to clarify the role of stringency factor.

Much of the data in the public databases comes from small scale studies which are challenging to integrate into a single resource for analysis as each dataset has been generated differently and comes with its own biases. Furthermore, many of the assays used to investigate interactions have their own biases. Many assays preferentially detect interactions between proteins that can be localized to the nucleus resulting in an underrepresentation of interactions for membrane bound or extracellular proteins. Only if all bacterial protein had been studies equally and were equally amenable to the assays used, can this sort of analysis be reliably performed.

We thank the reviewer for promoting discussion on this issue. Though we selected homogeneous datasets and carefully checked our methods to avoid technical biases, we acknowledge that additional controls can be used to strengthen our conclusions.

The datasets used are based on the yeast-two-hybrid (Y2H) method, one of the gold standards to define protein-protein interactions. As noted by the reviewer, proteins must localize to the nucleus to activate the transcription of the reporter gene. This can generate bias as some interactions may not be detected by Y2H. Unfortunately, there is no universal high-throughput method that can be applied to all proteins in the host-pathogen context. To the best of our knowledge, the dataset used is the most complete at present, containing enough data to get statistically meaningful results. However, in order to test for methodological biases, we used another source, the PHISTO database: http://www.phisto.org. This database is specific for host-pathogen interactions and reports data obtained by different methods, including Y2H, pull-down or affinity chromatography, among many others. Using this set of highly heterogeneous information, we reproduced the pipeline described in the manuscript and observed that similar conclusions can be drawn (Figure R3; p=0.037).

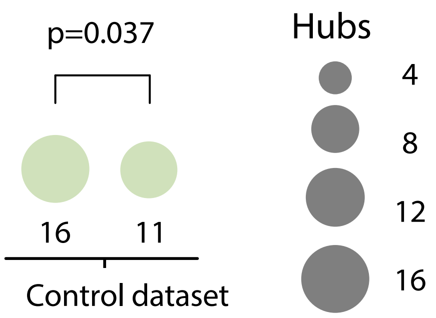

Figure R3. Effectors are significantly depleted of pathogen hubs. The control dataset confirms that the number of hubs observed is significantly lower than expected.

In our opinion, the methodologies and approaches used in our study are robust and based on homogeneous data. We used the same methods to analyze all species to ensure that they are comparable. Also, we validated our observations using a different dataset. Hence, we conclude that our analyses are meaningful and not strongly affected by experimental biases, as far as it is possible to test. Yet, as noted by the reviewer, we acknowledge potential limitations related to the Y2H technology. We describe them in the Materials and Methods section (pages 8-9) for the reader to have a better perspective of this method.

The same argument applies to the subsequent analysis of the HP and PP networks. The results shown in 1C could reflect subsets of proteins well studied in HP context but not PP (cluster 1), well studied in PP but not HP (cluster 2) and ones studied in both contexts and hence no difference if degree (cluster 3). It would be helpful for the reader if the authors provided a list of all interactions and their sources in a supplementary table.

We thank the reviewer for raising this point. When conducting the experiments, we did not observe any bias in this context. However, to better support our claim, we tested whether the amount of research related to each cluster may bias our study. We reasoned that if clusters C1 and C2 contain proteins that are highly studied (either in HP or PP context) compared to C3, we should see a difference in the amount of related literature. We mined for references in Pubmed citing proteins belonging to C1, C2 and C3 (both in *Y. pestis, B. anthracis and F. Tularensis* and other related bacteria such as *E. coli, P. aeruginosa* and *S. enterica*). We did not find any statistical differences between the three groups, suggesting that proteins are similarly studied, as depicted in Figure R4.

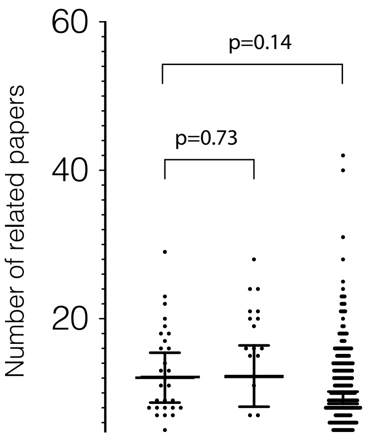

Figure R4. Literature analysis of clusters. The number of citations in Pubmed for each protein included in clusters C1, C2 and C3 is compared.

We would like to note that these clusters show specific structural properties (Figure 1D). These differences are independent from the presence of well-studied proteins and support our conclusions. Also, as suggested by the reviewer, we have added Supplementary Table 3, a list of all proteins included in each cluster.

The authors then proceed to study the amino acids which make up the interaction interfaces based on complexes available in PDB. Here again study bias could significantly impact the results and not all proteins or types of interactions are equally well represented in databases such as PDB. Additionally, it is unclear to what extent the same proteins were present in the BE, BB, and EE datasets.

To control for possible biases in the PDB database, we performed additional controls to strengthen our conclusions. The overlapping in BE, BB and EE datasets is very low as it is very difficult to find a protein crystallized in these two conditions. This excludes the possibility that certain entries are dominating the results. As mentioned in our report to Reviewer 1 (please see pages 1 and 2 in this letter), we did not find any bias in the interface size (computed as the number of interactions per molecule). Also, we applied a randomized analysis of our dataset to control the effect size and found similar results as those reported in our manuscript. We discuss the potential biases and limitations of the PDB database in the Materials and Methods section (Pages 8-9).

Finally, the authors explored a Y. pestis - host interactome. Again, it is unclear how comparable the Y-H interactome dataset is to the Y-Y or H-H datasets. Were these resources generated in comparable manners, using similar technologies and rigor, of similar relative sizes or completeness, and of equal quality? The observed overlap between Y-H and H-H networks may reflect technological and study biases rather than biological properties.

In this case, we compared the YH interactome to the YY or HH interactomes. Both, YY and HH interactomes were obtained from the same database (https://string-db.org) and the selection criterion was homogeneous (only experimentally validated interactions with a confidence score higher than 700), so we expect no bias in resource and data quality. The YY interactome has 3701 interactions for 3973 distinct protein-coding genes and the HH interactome has 18982 interactions for 19566 distinct protein-coding genes. Hence, the number of interactions per protein is similar and we do not expect a bias related to relative size. We acknowledge that database-related biases may occur when comparing the domain association overlap between YH-YY or YH-HH relative to YY-HH. Hence, we refrained to make any comparisons here. Completeness is a difficult metric to evaluate, as it is impossible to assess the true percentage of interactions that are detected in each organism. However, when comparing the results in *Y. pestis* with *B. anthracis* or *F. tularensis*, we observe similar percentages of shared domain associations between human-pathogen and human-human interactomes (21% in *Y. pestis*, 18% in *B. anthracis* and 17% in *F. tularensis*), suggesting that our estimates are sound.

MINOR POINTS

Fig 1a - the use of circles to show the number of hubs as effectors or non-effectors makes it is impossible to actually see the values being reported or really compare the differences. Why not just a bar plot?
We wanted to keep the same scale for all three species, and we reasoned that a dot plot would be simpler than a bar plot. Following the reviewer suggestion, we have added numbers in Figure 1A so the reader can compare them better.

Fig 2A The number of analyzed complexes doesn't match the text on page 4 or in the figure legend
We apologize for the mistake, the numbers reported in the text and figure legend have been corrected.

Fig 2B - did you control for the aa structure of the protein (or the fragment of the protein in PDB for which you had data) when calculating the enrichments in Interface, rim & surface)?
Yes, all percentages detailed in Figure 2B were controlled by the amino acid structure of the protein. We have added a note in the Figure legend to clarify this point.

Page 4, states "While some residues (Trp, Phe and Lys)" but looking at Fig 2B this can't be seen
The text mentioned by the reviewer accounts for differences observed between the eukaryotic and bacterial counterparts in EB complexes, which is described in Figure S2. We have added a mention to the figure to clarify the sentence.

Page 6 call out to Fig 1F, I think you mean 2F.
We apologize for the mistake, we have changed 1F by 2F.
