# Supplementary material for: Bacteria use structural imperfect mimicry to hijack the host interactome
Source: PLoS Comput Biol. 2020 Dec 4;16(12):e1008395. doi: 10.1371/journal.pcbi.1008395 (PMC7744059; doi:10.1371/journal.pcbi.1008395)
Supplement: Attachment — Submitted filename: Reponse_to_reviewers.pdf [file pcbi.1008395.s012.pdf]

Point-by-point response

Reviewer #1

The manuscript "Bacteria use Structural imperfect mimicry to hijack the host interactome" by Natalia de Groot and Marc Burgas focuses on understanding how pathogen proteins bind their host counterparts, which is important to gain insight on how bacteria can infect, survive and proliferate inside cells. Overall, the proposed ideas are interesting, and can be of interest to the readers. But I still have some reservations for this manuscript, which I would like the authors to consider, if a revision is permitted.

We thank the reviewer for considering our paper of interest to the scientific community. We have made every effort to clarify several concepts in our manuscript, particularly, the idea of molecular mimicry. As stated in our previous response letter, we are not confident that an analysis of non-pathogenic complexes can be undertaken at this point. Moreover, we believe that such analysis could be misleading due to incomplete knowledge of pathogenic mechanisms. Finally, as suggested by the reviewer, we have further described our BE dataset and investigated whether proteins in this set have a substantial effect in pathogen fitness and whether they are differentially regulated upon infection. We hope that the revised version will meet the expectation of this reviewer for publication.

1. I am not sure I fully appreciate the idea of mimicry. As I understand, mimicry means pathogens mimic the host protein, but the paper looks at it in terms of individual amino acids, and to say that positive and negative charges interact doesn't seem like enough to conclude the pathogen is trying to mimic the host (short sequences or confirming host interaction sites are the same would be better).

We thank the reviewer for sharing his/her views on our manuscript. We would like to clarify in this response letter the two points raised here by the reviewer: the concept of molecular mimicry and the nature of our analysis.

The concept of mimicry in host-pathogen interactions

We would like to stress that we do not coin in this manuscript the concept of mimicry. The idea of *molecular mimicry* was proposed by Raymond Damian in 1964[1]. In his paper, Prof. Damian noticed that similar antigens expressed by infectious agents and their human hosts may facilitate microbes to avoid the host immune response. Since then, the term molecular mimicry has been extensively used in the field, as detailed in our manuscript (page 5). Profs. Nels Elde and Harmit Malik provide an excellent review on this topic[2]. Many authors have addressed this question in terms of sequence similarity and co-evolution, as suggested by the reviewer [3,4]. However, the use of primary structure can be limited as structural mimicry can happen with very low, if any, sequence similarity. There are even some examples where structural similarity can be of no avail. This is the case of invasin, a protein from *Yersinia* that

[1] Damian, R. T. (1964). Molecular mimicry: antigen sharing by parasite and host and its consequences. *The American Naturalist*, *98*(900), 129-149.
[2] Elde, N. C., & Malik, H. S. (2009). The evolutionary conundrum of pathogen mimicry. Nature Reviews Microbiology, 7(11), 787-797.
[3] Hagai, T., Azia, A., Babu, M. M., & Andino, R. (2014). Use of host-like peptide motifs in viral proteins is a prevalent strategy in host-virus interactions. Cell reports, 7(5), 1729-1739.
[4] Stebbins, C. E., & Galán, J. E. (2001). Structural mimicry in bacterial virulence. Nature, 412(6848), 701-705.

binds to human integrin and helps the pathogen to invade the host epithelium [5]. Invasin can bind to β1 integrins with a much higher affinity than extracellular matrix ligands, such as fibronectin [6]. Despite the lack of sequence and structural homology between them, fibronectin and invasin share similar binding sites [7]. Given this scenario, we decided to use network analysis to uncover the molecular signatures of host-pathogen PPIs.

Why use network analysis to investigate interactions?

Residue Interaction Networks (RINs) represent amino acid residues as nodes in a network [8]. If two residues interact (based on spatial distance) they are connected by edges between them. Hence, these networks capture inter-residue interactions (Figure 1 in this letter). Several studies support that residue network topology can provide insight into the structure-function relationship and have been used to uncover key residues for protein stability and dynamics [9], enzymatic activity [10] or signal transduction [11].

As noted in the manuscript, we did not consider just the composition of amino acids and the mere fact that positive and negative charges interact. We compared the connectivity profiles of protein complexes and used them as interaction fingerprints (Figure 1 in this letter provides a visual summary of BE interactions). Several conclusions can only be drawn from such analysis, as explained in the manuscript. First, we analysed the overall connectivity degree (measured as the sum of all contacts in complexes, Figure 2D in the main manuscript). Even though BE and BB complexes show similar amino acid composition at the interface, the number of contacts associated to each residue is completely unrelated. Conversely, the connectivity degree is similar between BE and EE complexes, which reinforces the idea of mimicry from a network point of view. However, what is most interesting to us, is that the configuration of these interactions has noticeable differences that are relevant for understanding the nature of interfaces. For example, interacting Trp is mostly targeted at the eukaryote site whereas Tyr is the opposite. In our paper, we describe the case of anion-pi interactions as a conspicuous example of a distinctive BE interaction signature.

Hence, we analysed the complex interfaces in the view of contact networks, not on amino acid composition or the physicochemical nature of interactions. The differences observed represent changes in the interaction pattern, suggesting that bacteria use, preferentially, a particular set of interactions, which is different from the eukaryote set. Hence, we conclude that mimicry between BE and EE interfaces is imperfect.

We have added several lines in the methods section to justify the use of RINs for the analysis of host-pathogen PPIs.

---

[5] Andrea, M., & Isberg, R.R. (1997) Invasin-dependent and invasin-independent pathways for translocation of Yersinia pseudotuberculosis across the Peyer's patch intestinal epithelium. *Infection and immunity* 65(8): 3412-3421.

[6] Tran Van Nhieu, G., and Isberg, R.R. (1993) Bacterial internalization mediated by beta 1 chain integrins is determined by ligand affinity and receptor density. *The EMBO Journal* 12(5): 1887-1895.

[7] Hamburger, Z. A., Brown, M. S., Isberg, R. R., & Bjorkman, P. J. (1999). Crystal structure of invasin: a bacterial integrin-binding protein. *Science*, 286(5438), 291-295.

[8] Shcherbinin, D., & Veselovsky, A. (2019). Analysis of protein structures using residue interaction networks. In Structural Bioinformatics: Applications in Preclinical Drug Discovery Process (pp. 55-69). Springer, Cham.

[9] Rao, F., & Caflisch, A. (2004). The protein folding network. *Journal of molecular biology*, 342(1), 299-306.

[10] Amitai, G., Shemesh, A., Sitbon, E., Shklar, M., Netanely, D., Venger, I., & Pietrokovski, S. (2004). Network analysis of protein structures identifies functional residues. *Journal of molecular biology*, 344(4), 1135-1146.

[11] del Sol, A., Fujihashi, H., Amoros, D., & Nussinov, R. (2006). Residues crucial for maintaining short paths in network communication mediate signaling in proteins. *Molecular systems biology*, 2(1), 2006-0019.

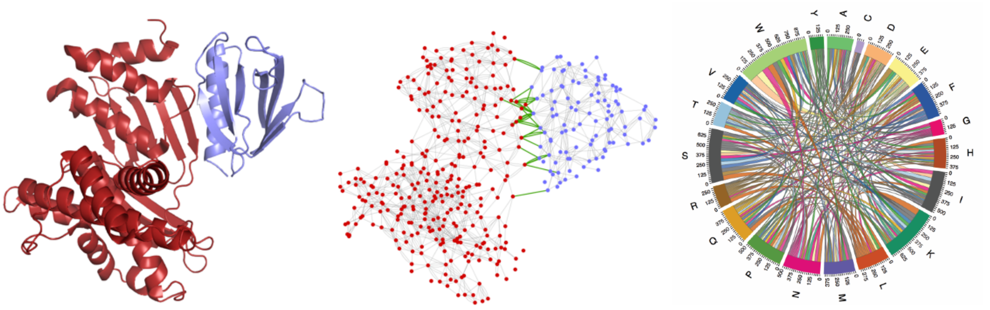

Figure 1. Representation of protein complexes as residue interaction networks. A protein complex (left panel, SifA-PlekHM2 in PDB 3HW2) can be represented as a network of contacts (central panel, SifA residues in blue, PlekHM2 residues in red, intra-chain contacts in grey and inter-chain contacts in green). The inter-chain contacts in complexes can be calculated and summarized to obtain (right panel, circos plot summarizing all BE contacts).

2. Though the interactome of BE is validated, which is more similar to EE than BB, authors need to show any non-pathogenic BE won't have the same similarity to EE of pathogenic BE.
3. If the interactome database for non-pathogenic BE is scarce then the authors should not discuss the pathogenicity as a mimicry of EE. This undermines the importance of the pathogenicity validation besides the analytical resemblance of BE to EE.

As mentioned in our previous letter, there is a lack of validated data to perform such analysis. This statement is also supported by other authors. In a recent manuscript posted in bioRxiv, J. Beltrán and I. Brito analyse the host-microbiome protein-protein interactions [12]. In this paper, the authors predict interactions between 478 bacterial gene clusters and 2,140 human proteins. They used BLASTP to match protein sequences to crystal structures in the PDB, with proper filters by sequence identity and coverage. They found only 20 cocrystal structures that provide interface information for only 18 protein pairs. From all these cases, they could analyse only 6 complexes. The authors concluded that there is not enough information in the databases to perform structural prediction at large scale for the commensal human microbiome.

Still, even if such analysis could be done, the comparison of pathogenic and non-pathogenic interactions could be misleading and would not be suitable for comparison.

Identification of pathogenic and non-pathogenic proteins

Caution must be taken to classify bacteria, and their proteins, as non-pathogenic. The division between these two concepts is diffuse and can depend on physiological conditions. First, because bacteria can switch between pathogenic and non-pathogenic states depending on the environment. Second, because non-pathogenic and pathogenic bacteria can share proteins that, only under appropriate conditions, can behave as pathogenic.
Streptococcus pneumoniae is a remarkable example of the first case. *S. pneumoniae* is a commensal bacterium that is present in the nasal microbiota [13]. However, in certain conditions, it can move to the lungs causing severe pneumonia in elderly people or immunocompromised patients. Other studies have observed that particular mutations in some genes can convert non-pathogenic bacteria into pathogenic. This is the case of

[12] Beltrán, J.F., & Brito, I.L. (2020) Host-microbiome protein-protein interactions reveal mechanisms in human disease. *bioRxiv*: 821926.
[13] Weiser, J.N., Ferreira, D.M. & Paton, J.C. (2018) Streptococcus pneumoniae: transmission, colonization and invasion. *Nature Reviews Microbiology* 16(6): 355-367.

Escherichia coli where mutations in the LPS transporter [14] or the histone-like protein HU [15] in non-pathogenic strains can lead to high virulent or invasive strains, respectively.

A given protein can be classified as pathogenic if it interferes with the host homeostasis and/or is associated with the development of infection. Hence, proteins that, when deleted or mutated, change the progression of infection can be univocally classified as pathogenic. The reverse reasoning, however, is not valid. We can find an example in hemolysin or curli fibres. These proteins are virulence factors that help bacteria to lyse cells by inducing membrane pores or induce aggregative phenotypes that help the formation of biofilms in host tissues. However, these genes can be present both in pathogenic and non-pathogenic bacteria. In non-pathogenic bacteria they are quiescent, but its expression can be triggered under certain conditions, increasing the virulence of these otherwise non-pathogenic strains. Mutations in protein HU, as mentioned before, can generate virulence in non-pathogenic strains by inducing the expression of certain genes [16]. Another example is the ecotin protein. The aforementioned study by Beltrán et al. identified the complex between the ecotin family and blood coagulation factor Xa. Though this protein was identified in the human microbiota, ecotin is also classified as a virulence factor that blocks multiple complement-dependent and independent microbicidal activities [17].
In conclusion, we are not convinced that non-pathogenic BE complexes can be unequivocally delineated, and we believe that such comparison, at least with the present amount of data, can be flawed by incomplete knowledge.

Comparison between pathogenic and non-pathogenic protein complexes
As noted in the previous paragraph, it can be difficult to find non-pathogenic BE complexes without a shadow of a doubt. But, even in the case where we could find an appropriate set of complexes, our conclusions would be independent of these findings. We do not claim that proteins are pathogenic because of imperfect mimicry but that bacteria use imperfect mimicry to interact with the host and hijack the interactome. In other words, we suggest that bacteria use specific interaction patterns to interact with the host by means of interface mimicry. The fact that non-pathogenic interactions use (or do not use) mimicry to interact with their host counterparts, does not invalidate or undermine our conclusions.
We can only speculate at this point what could happen for non-pathogenic proteins. They may use its own version of mimicry, with different signatures than the ones detailed in our paper due to a different evolutionary pressure. Commensal bacteria do not generally inject proteins into host cells to modify the interactome and behave mostly at the extracellular level, changing the number of metabolites, etc. Hence, the kind of interactions between organisms might be probably different. Though this would be an interesting question to address, it is beyond the scope of our manuscript.

---

[14] Kaito, C., Yoshikai, H., Wakamatsu, A., Miyashita, A., Matsumoto, Y., Fujiyuki, T., ... & Sekimizu, K. (2020). Non-pathogenic Escherichia coli acquires virulence by mutating a growth-essential LPS transporter. *PLoS Pathogens*, 16(4), e1008469.
[15] Koli, P., Sudan, S., Fitzgerald, D., Adhya, S., & Kar, S. (2011). Conversion of commensal Escherichia coli K-12 to an invasive form via expression of a mutant histone-like protein. *MBio*, 2(5).
[16] Kar, S., Edgar, R., & Adhya, S. (2005). Nucleoid remodeling by an altered HU protein: reorganization of the transcription program. *Proceedings of the National Academy of Sciences*, 102(45), 16397-16402.
[17] Nagy, Z.A., Szakács, D., Boros, E., Héja, D., Vígh, E., Sándor N, … & Pál, G. (2019) Ecotin, a microbial inhibitor of serine proteases, blocks multiple complement dependent and independent microbicidal activities of human serum. *PLoS pathogens* 15(12): e1008232.

4. Authors claim that BE interactome includes pathogenic effectors, which needs to be substantiated in detail on the related mechanism from the dataset, or verify from the existing antimicrobial effect on disturbing the interfaces of BE.

We agree with the reviewer that a more detailed description of the BE dataset would strengthen the conclusions of our manuscript. A description of the proteins in the BE dataset has been added in Supplementary Table 1. Proteins in the BE set include effectors, toxins, adhesins, hemolysins and other proteins involved in bacterial pathogenesis.
To further validate the role of these proteins in infection, we used blastp against the BacFitBase database [18] to predict the fitness score associated with each protein (Figure 2 in this letter). Indeed, the fitness defect associated to these proteins is larger than a null effect (p=0.0005, negative values indicate a reduction on pathogen fitness). Hence, we demonstrated that the BE dataset is indeed associated with pathogenesis. Moreover, if we analyse the mRNA expression data using the DualSeqDB database [19], proteins belonging to the BE dataset are significantly upregulated in infection (p<0.0001) suggesting, once again, its implication in bacterial pathogenesis. We have added this figure in the manuscript, under the Supplementary Information section.

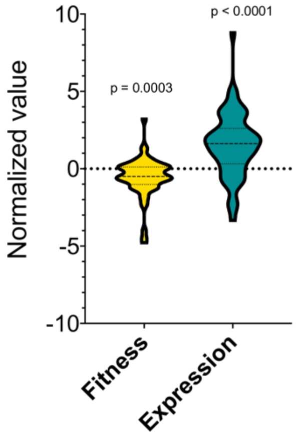

Figure 2. Fitness impact and differential expression of bacterial proteins belonging to the BE set.

5. With no pathogenicity, the resemblance of BE to EE might be a common phenomena to share the evolutionary residue when overwhelming number of proteins being interacting.

We truly apologize but we do not fully understand what the reviewer means with this comment. We observed, as detailed in Figure 2 (main manuscript), that the composition of amino acids is similar between BE and EE interfaces. Hence, the evolutionary effect on residue composition cannot explain the differences found, even when a large number of proteins is considered. We have also demonstrated in the previous point that proteins in BE dataset are indeed related to pathogenesis. We hope that with the data presented in this response letter, the reviewer will find our results conclusive.

6. I appreciate the innovative idea put forward in the data mining process and analysis. But still the strategies needs to be detailed with respect to the development of antimicrobial agents from BE, if the claim is true.

We believe that imperfect mimicry may be used in our advantage to develop new antimicrobials. If an interaction is required for the pathogen to infect the host, blocking this interaction would help to stop or delay the infection. Hence, compounds designed to interfere with such host-pathogen interactions would behave as antimicrobials. This idea has been suggested by us previously [20] but also by other authors [21,22]. The results presented in this

---

[18] Rendón, J. M., Lang, B., Tartaglia, G. G., & Burgas, M. T. (2020). BacFITBase: a database to assess the relevance of bacterial genes during host infection. *Nucleic Acids Research*, 48(D1), D511-D516.
[19] Rendón, J. M., Lang, B., Tartaglia, G. G., & Burgas, M. T. (2020). DualSeqDB: The host-pathogen dual RNA sequencing database for infection processes. *In revision*. Website: http://www.tartaglialab.com/dualseq/
[20] Asensio, N.C., & Torrent, M. (2017) Centrality in the host–pathogen interactome is associated with pathogen fitness during infection. *Nature communications* 8(1): 1-6.
[21] Carro, L. (2018) Protein–protein interactions in bacteria: a promising and challenging avenue towards the discovery of new antibiotics. *Beilstein Journal of Organic Chemistry* 14(1): 2881-2896.
[22] Munguia, J., & Nizet, V. (2017) Pharmacological targeting of the host–pathogen interaction: alternatives to classical antibiotics to combat drug-resistant superbugs. *Trends in pharmacological sciences* 38(5): 473-488.

paper conceptually takes us one step further in this effort. We propose that bacteria use imperfect mimicry to interact with host proteins. In other words, the network of interactions in host-pathogen PPIs is different from the own host network. Because of this, we could think of compounds, either peptidomimetics or small molecules, that interact with the bacteria interface without disrupting the host complexes. Our observations suggest that such compounds might have therapeutic potential as the interaction with their host targets would be weaker compared with their bacteria counterparts.
We acknowledge that a more detailed discussion in the manuscript would be helpful to further develop the concept. For this reason, we have extended the last paragraph in the discussion to better describe the implications of imperfect mimicry in the development of potential bacteria PPI inhibitors.

Overall, I think the manuscript is not fully developed with lots of lingering questions still. I feel authors claim on the "mimicry of EE by pathogen BE" sounds nebulous as there is no proper comparison performed with non-pathogenic BE due to lack of data. As well, there is no elaboration on how antimicrobial development would be utilized by data mining, including for very specific rim, core and individual AA interaction in BE. Without these details and additional analyses, I think the findings from this study may be overstated.

We thank the reviewer for sharing his/her thoughts about our manuscript. We sincerely hope to have addressed all the concerns in this letter and that the revised version will meet the expectation of this reviewer for publication.
